# Beneficial effects of *Vigna angularis* extract in osteoporosis and osteoarthritis

**DOI:** 10.1002/fsn3.1944

**Published:** 2020-10-20

**Authors:** Hyung Jin Lim, Sang‐Ik Park, Seon Gyeong Bak, Sun Hee Cheong, Soyoung Lee, Young‐Bin Baek, Chang‐Min Lee, Kang Min Lee, Seung Woong Lee, Seung‐Jae Lee, Mun‐Chual Rho

**Affiliations:** ^1^ Immunoregulatory Material Research Center Korea Research Institute of Bioscience and Biotechnology Jeongeup‐si Korea; ^2^ College of Veterinary Medicine Chonnam National University Gwangju‐si Korea; ^3^ Department of Marine Bio Food Science Chonnam National University Yeosu‐si Korea; ^4^ Department of Molecular Biology Chonbuk National University Jeonju‐si Korea

**Keywords:** monosodium iodoacetate, osteoarthritis, osteoporosis, ovariectomy, *Vigna angularis*

## Abstract

In Asia, *Vigna angularis* (azuki bean) has been used as a traditional medicine to treat various diseases because of its biological properties. Osteoarthritis (OA) and osteoporosis (OP) are common regenerative bone diseases that are characterized by deterioration of joint and bone structure. In this study, we evaluated the effects of *Vigna angularis* extract (VAE) on monosodium iodoacetate (MIA)‐induced OA and ovariectomy (OVX)‐induced OP models. In the MIA‐induced OA results, severe OA was alleviated by the administration of VAE. Extensive local damage in the cartilage and hemorrhagic and edematous of surrounding tissues were decreased by VAE treatment. Articular cartilage was almost intact except for a focal mild abrasion, and the surface was glistening, similar to that of the normal joint. In the OVX‐induced OP results, bone mineral content (BMC) and bone mineral density (BMD) were recovered by VAE treatment, and it improved the microstructures of bone. These results show that VAE could inhibit OA and OP symptoms.

## Graphical abstract

We investigated the regulatory effects of bone diseases of ethanolic extract from Vigna angularis (azuki bean). Vigna angularis (azuki bean) could be serve as natural bone‐regulatory material.

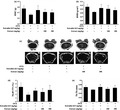

## 1 INTRODUCTION

*Vigna angularis* (azuki bean) is an annual legume crop grown through East Asia (Kang et al., 2015) *V. angularis* has been used as a traditional medicine in Korea, China, and Japan to treat infection, inflammation, and edema of the kidney and bladder, and various biological properties have been reported, such as antioxidative, antihypertensive, and anti‐inflammatory activities (Oh et al., 2014; Hori, Sato & Hatai, 2006). Phenolic acids, polyphenols, triterpenoids, and flavonoids are known to be phytochemical components of *V. angularis* (Lee et al., 2017).

Osteoarthritis (OA) is a common degenerative arthritis that is characterized by progressive deterioration of articular cartilage, osteophyte formation, cartilage calcification, and pain (Eyre, 2001; Goldring, 2012; Sokolove & Lepus, 2013). In the progression of OA, inflammatory mediators are observed in the joints, and these mediators induce extracellular matrix (ECM) degradation and MMP synthesis, which increase cartilage degradation (Hashimoto et al., 1998; Liu‐Bryan, 2013). The residue of ECM and cartilage degradation bind and activate pattern recognition receptors (PRRs) and induce proinflammatory cytokine production in chondrocytes, macrophages, and fibroblast‐like synoviocytes (Pelletier et al., 2001; Piccinini & Midwood, 2010). There are several options for treating OA, including the administration of anti‐inflammatory drugs (Sinusas, 2012). Corticosteroids and nonsteroidal anti‐inflammatory drugs are commonly prescribed as OA medications (Johnston & Budsberg, 1997). However, these drugs may have side effects such as vomiting, gastrointestinal bleeding, gastrointestinal toxicity, and renal toxicity (Lee et al., 2019; Wang et al., 2011). Thus, traditional herbal medicinal sources with no or minor side effects have been widely investigated.

Osteoporosis (OP) is a common metabolic bone disease characterized by low bone mass, which is due to an imbalance of bone formation and bone resorption (Ste‐Marie, 1995). Osteoclasts (OCs) and osteoblasts (OBs) are regulators of bone resorption and bone formation. Receptor activator of NF‐κB ligand (RANKL) and osteoprotegerin (OPG) are released from OBs (Neyro et al., 2011). RANKL binds receptor activator of NF‐κB (RANK) and activates mitogen‐activated protein kinase (MAPK) and nuclear factor‐κB (NF‐κB) signaling and differentiates hematopoietic precursor cells to OCs (Abu‐Amer, 2013). OPG binds to RANKL and inhibits the binding of RANKL to RANK. Estrogen deficiency in perimenopausal women causes excessive bone resorption by osteoclasts and results in osteoporosis (Fitzpatrick, 2006). Estrogen inhibits proinflammatory cytokines, such as IL‐1β, IL‐6, and TNF‐α, which promote osteoclast differentiation, and increases TGF‐β and OPG, which inhibit osteoclast differentiation (Brincat et al., 2014; Riggs, 2000).

In this study, we evaluated the effect of *V. angularis* extract (VAE) on a monosodium iodoacetate (MIA)‐induced osteoarthritis and ovariectomy (OVX)‐induced osteoporosis animal model. It would show its potential as an alternative to the treatment of OA and OP.

## 2 MATERIALS AND METHODS

### 2.1 *Vigna angularis* extraction

*V. angularis* (20 kg) was pulverized until it was smaller than 10 mesh size and extracted with 95% EtOH (100 L) at 50°C overnight using the circulation method. After filtering (1‐micrometer filter paper) the extract in vacuo, the filtrates were evaporated under reduced pressure to generate the first ethanol extract (476 g). This extract was used for in vivo experiments.

### 2.2 Animals

Twenty‐four twelve‐week‐old female Wistar rats and thirty six‐week‐old female C57BL/6 mice were used in this study. The animals were kept in an air‐conditioned animal room at 22°C and were given tap water and a basal diet. The animals were acclimated for 1 week before use. All animal experiments were performed according to guidelines for animal handling and welfare in our facilities. Institutional Animal Ethics Committee approval for the experimental protocol was obtained before initiation of the study by the Institutional Animal Care and Use Committee (IACUC).

### 2.3 Induction of osteoarthritis

OA was induced through a single intra‐articular injection of monosodium iodoacetate (MIA), as previously described (Gerwin et al., 2010). The rats were randomly divided into 4 groups (*n* = 6 rats per group): saline injected and saline orally administered rats (SHAM), MIA injected and saline orally administered rats (MIA), MIA injected and VAE (50 or 100 mg/kg) orally administered rats. MIA (cat. #I2512; Sigma) was dissolved in 30 μL of sterile saline, and 2 mg of MIA was administered under ketamine anesthesia. Both knees were shaved and disinfected. Then, saline or MIA was injected at the center of the right knee. After MIA injection, saline and VAE were orally administered every day for 4 weeks. To evaluate improvements in OA, the rats were sacrificed, and hind limbs were removed.

### 2.4 Induction of osteoporosis

OP was induced through ovariectomy, as previously described (Kim et al., 2016). Mice were randomly divided into 5 groups (*n* = 6 mice per group): sham‐operated mice (SHAM), OVX mice orally administered saline (OVX), OVX mice orally administered 0.5 mg/kg (E2), and OVX mice orally administered 100 or 300 mg/kg VAE. After stabilization, sham operation or bilateral ovariectomy was performed under ketamine anesthesia. Then, the mice were maintained without any treatment for 6 weeks until the occurrence of bone loss. After 6 weeks, the mice were orally administered saline, estradiol, and extract every day for 6 weeks. To evaluate improvement in OP, the mice were sacrificed, and femurs were collected for microcomputed tomography (micro‐CT) analysis.

### 2.5 Histopathological analysis of the MIA‐induced osteoarthritis rat model

The animals were medicated with ketamine and sacrificed by cervical dislocation. Both hind limbs were immediately disarticulated at the hip joint, and all knees were fixed in 10% neutral‐buffered formalin for 72 hr and decalcified using an EDTA solution (cat. #324506; Sigma) for 4 weeks. The right and left knees were excised sagittally and frontally, respectively, to evaluate the histopathological changes in the knee joints. Specimens were deacidified in 5% sodium sulfate solution for 72 hr, dehydrated in 100% ethanol after being washed with water, and embedded in paraffin wax. The decalcified sagittal and frontal plane paraffin specimens were prepared according to the Osteoarthritis Research Society International (OARSI) recommendations described by Gerwin et al. (2010). The 3‐μm‐sectioned slides were stained separately with hematoxylin and eosin (H&E) and 0.1% Safranin‐O/Fast Green for 5 min. Then, the slides were sequentially dehydrated in 70%, 80%, 90%, and 100% ethanol. Finally, the sections were cleared in xylene. A light microscope and a digital camera (DFC290; Lieca Corporation) were used to capture and evaluate the histopathological features of the articular cartilage and the infrapatellar fat pad (IFP).

### 2.6 ELISA of synovial fluid of the MIA‐induced osteoarthritis rat model

IL‐6 and TNF‐a cytokine levels in synovial fluid were analyzed by rat IL‐6 and TNF‐α ELISA kits (cat. #R6000B, RTA00; R&D systems) following the manufacturer's instructions. In brief, synovial fluid was incubated in capture antibody precoated wells for 2 hr. Then, the wells were washed and incubated with horseradish peroxidase (HRP)‐conjugated detection antibody for 2 hr. Finally, the wells were incubated with 3,3',5,5'‐tetramethylbenzidine (TMB) substrate for 30 min, and stop solution was added. The absorbance was determined by a microplate ELISA reader.

### 2.7 Dual energy X‐ray absorptiometry of the OVX‐induced osteoporosis mouse model

At the end of the osteoporosis experiment, bone mineral content (BMC) and bone mineral density (BMD) of the total body area of mice were evaluated using GE Lunar PIXImus2 Dual energy X‐ray absorptiometry (GE Healthcare) following the manufacturer's instructions.

### 2.8 Microcomputed tomography analysis of the OVX‐induced osteoporosis mouse model

The mouse femurs were fixed in 10% formaldehyde and stored in PBS. To evaluate trabecular bone analysis, a SkyScan 1076 micro‐CT scanner (BrukermicroCT) was used. Scans were conducted using an 88 kV source voltage and a 112 μA source current with 9 μm resolution. The trabecular bone volume per tissue volume (Tb. BV/TV), trabecular thickness (Tb.Th.), and three‐dimensional images of the femur were analyzed using the Nrecon®, CTAn®, and CTVol® software programs.

### 2.9 Statistical analysis

The histopathological data of the MIA‐induced OA model were scored according to the histopathological scoring system described by Kobayashi et al. (2003), which was nonparametric in nature. Accordingly, the Kruskal–Wallis test was used to compare more than two groups, which is parallel to the ANOVA test in the case of parametric data, and the Mann–Whitney *U* test was used to compare differences between two groups, which is parallel to the Student's *t* test in the case of parametric data. For statistical analysis of CIA‐induced OP data, statistical significance was determined by one‐way ANOVA followed by Tukey's test for multiple comparisons.

## 3 RESULTS AND DISCUSSION

### 3.1 Histopathological analysis of the MIA‐induced osteoarthritis rat model

**FIGURE 1:**
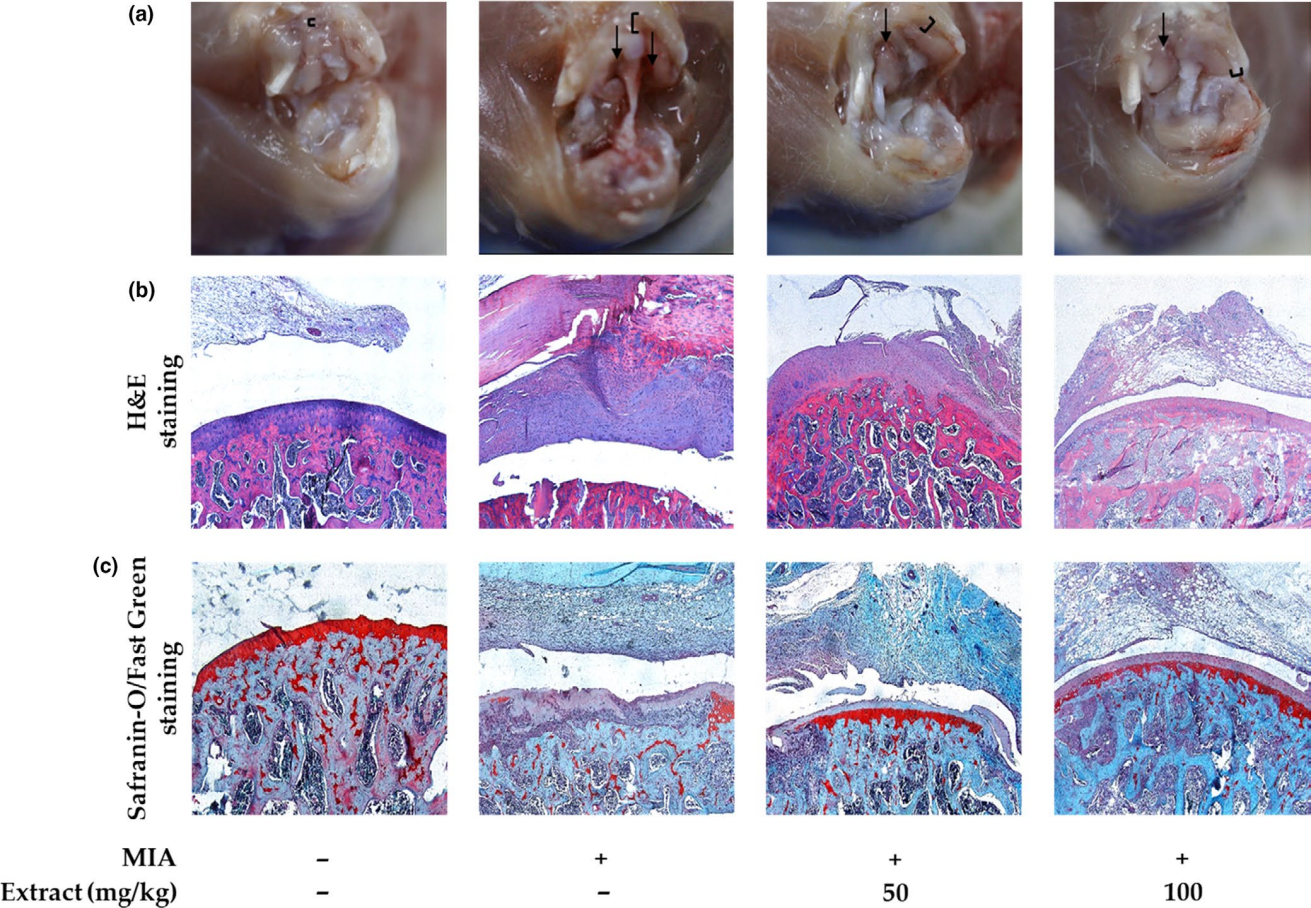
Gross observations (a) and histopathological features of knee joint tissues in MIA‐induced rats. Representative photographs of knee joint tissues stained with H&E (b) or Safranin‐O/Fast Green (c). Ι; the width of the synovium. Arrows: damaged articular cartilage (from left to right: normal control; osteoarthritis (OA)‐induced group treated with vehicle; OA‐induced group treated with 50 mg kg^‐1^ day^‐1^ VAE; and OA‐induced group treated with 100 mg kg^‐1^ day^‐1^ VAE)

**FIGURE 2:**
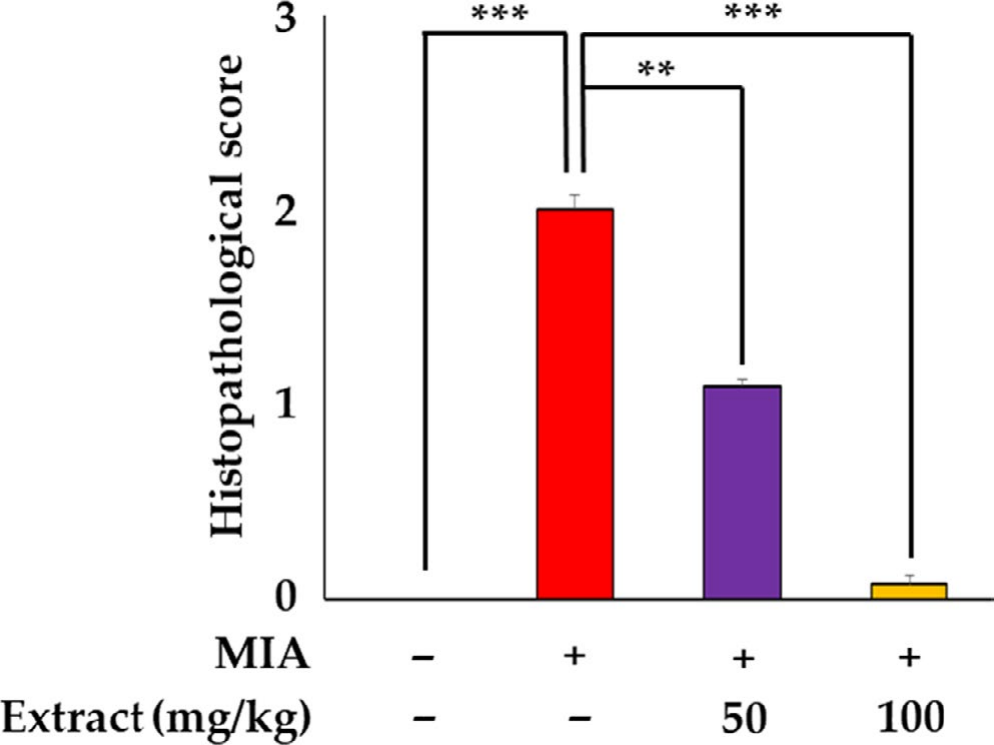
Histopathological scores of MIA‐induced rats. The values are presented as the means ± *SD* **p < .01, ***p < .001

In this study, the rats were sacrificed, and the knee joints were evaluated histologically to determine the severity of inflammation and cartilage damage using H&E and Safranin‐O/Fast Green staining. Gross observations of the injected right knee joints with or without 4 weeks of oral administration of VAE were performed. Normal morphology of the synovium and articular surface was observed with intact cartilage. Severe OA was induced 4 weeks after MIA injection and showed prominent thickening of the synovium and extensive local damage in the cartilage (Figure 1a). These histological features demonstrate that VAE attenuated the severity of MIA‐induced OA in rats. The surrounding tissues were hemorrhagic and edematous. MIA‐induced OA in the knee join was alleviated by the administration of VAE. The synovial thickness was reduced, and the articular cartilage seemed fairly normal. The protective effect of VAE on OA showed more efficacy at a higher dose. Articular cartilage was almost intact except for a mild focal abrasion, and the surface was glistening, similar to that of the normal joint. The MIA group exhibited severe arthritis with multiple, locally extensive infiltrations of macrophages and fibroblasts in the synovium of the knee joint, and the cartilage was severely degenerated and necrotic, showing an irregular surface (Figure 1b). In parallel, chondrocytes were severely damaged, resulting in a significant loss of ECM in the cartilage, which was confirmed by Safranin‐O staining, compared to those of the vehicle‐injected group (Figure 1c). Next, we measured the histopathological score (Table S1, Figure 2) based on three pathological grades: + (mild, score 1), ++ (moderate, score 2), and +++ (severe, score 3) (Colombo et al., 1983). Interestingly, treatment with MIA induced thickening of the subchondral bone and an increase in the ratio of trabecular bone to bone marrow (data not shown). The MIA resulted in moderate to severe pathology in the synovium, cartilage, and subchondral bone compared to those of the normal control. Treatment with 50 mg/kg VAE efficiently ameliorated pathological changes, and 100 mg/kg VAE markedly protected against OA (Figure 2).

### 3.2 Analysis of synovial fluid inflammatory cytokines in the MIA‐induced osteoarthritis rat model

**FIGURE 3:**
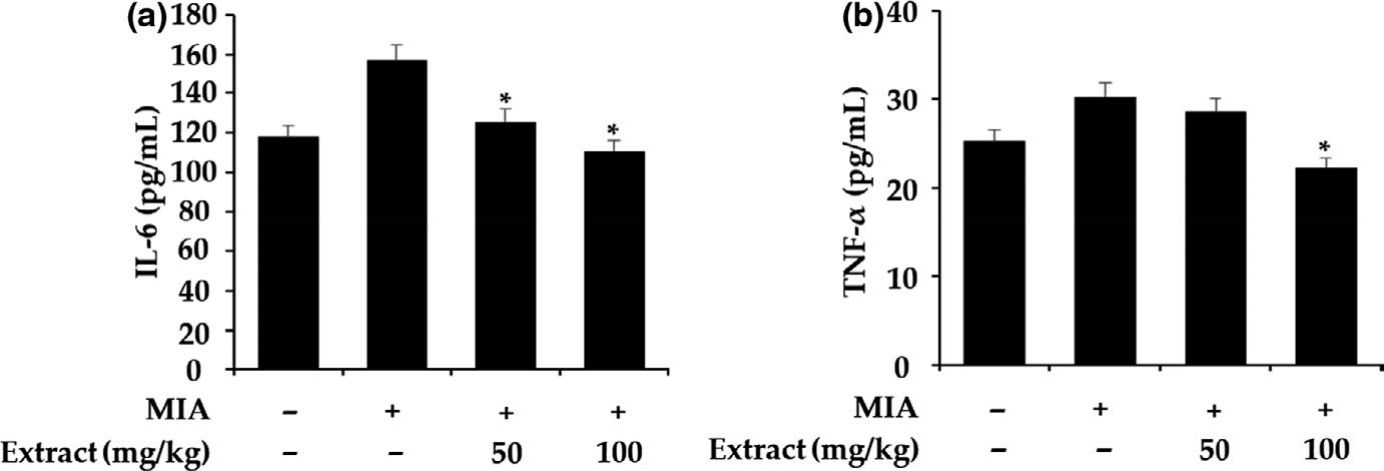
Proinflammatory cytokine levels in the synovial fluid of MIA‐induced rats. IL‐6 (a) and TNF‐α (b) concentrations in synovial fluid were measured by ELISA. The values are presented as the means ± *SD*. *p < .05, **p < .01 compared with only the MIA group

Next, we analyzed IL‐6 and TNF‐α levels in the synovial fluid and found significant decreases in both cytokines of VAE‐treated animals (Figure 3). In a previous study, we reported an inhibitory effect of *V. angularis* ethanol extract on IL‐6‐induced signal transducer and activator of transcription 3 (STAT3) activation (Oh et al., 2014). STAT3 is involved in various inflammatory diseases and mediates the inflammatory response. Many studies have developed therapeutic agents for inflammatory diseases by inhibiting STAT3 phosphorylation (Miyoshi et al., 2011; Yu et al., 2009). It has been reported that phosphorylated STAT3 levels are increased in chondrocytes from OA patients compared with those from normal controls (Hayashi et al., 2015). The therapeutic effect of inhibiting STAT3 phosphorylation in an MIA‐induced OA rat model has also been reported (Lee et al., 2018). We previously conducted studies showing that VAE inhibited STAT3 by VAE. However, more research needs to be done in the context of OA, and while the efficacy of VAE has been confirmed in animal models, the study of mechanisms through biomarkers is essential.

### 3.3 Dual energy X‐ray absorptiometry and micro‐CT analysis of the OVX‐induced osteoporosis mouse model

**FIGURE 4:**
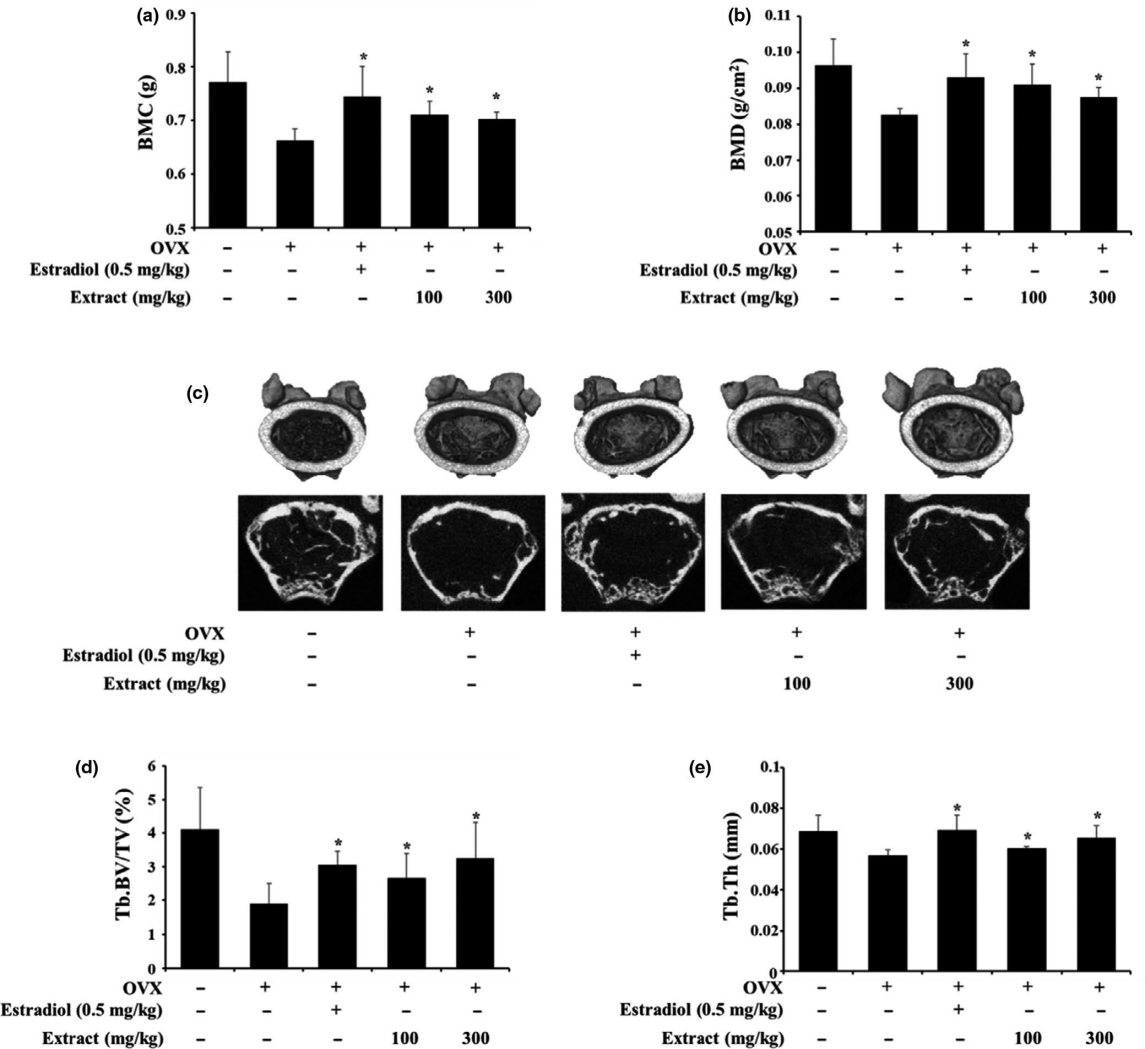
Dual energy X‐ray absorptiometry and microcomputed tomography analysis of OVX‐induced mice. Bone mineral content (BMC) (a) and bone mineral density (BMC) (b) of OVX‐induced mice were measured by dual energy X‐ray absorptiometry. Micro‐CT images of the proximal femurs (c), trabecular bone volume/tissue volume (Tb. BV/TV) (d), and trabecular thickness (Tb. Th) (e) were obtained using micro‐CT. The values are presented as the means ± *SD*. *p < .05, **p < .01 compared with only the OVX operation group

To evaluate the effect of VAE on the OVX‐induced osteoporosis mouse model, we performed dual energy X‐ray absorptiometry (DEXA) and micro‐CT analysis. In the DEXA results, only the OVX‐operated group showed decreased BMC and BMD, which are indicators of osteoporosis and fracture risk. However, treatment with 100 mg/kg and 300 mg/kg VAE significantly increased BMC and BMD compared with only the OVX‐operated group (Figure 4a and b). In the micro‐CT results, only the OVX‐operated group showed discontinuity of cancellous bone and decreased trabecular bone thickness (Figure 4c and e). Furthermore, Tb. BV/TV was decreased in only the OVX‐operated group (Figure 4d). VAE treatment recovered bone microstructures and Tb. BV/TV value compared with only the OVX‐operated groups (Figure 4c‐d). As we mentioned above, VAE inhibits the IL‐6/STAT3 signaling pathway. In bone metabolism, IL‐6/STAT3 is involved in bone resorption of osteoclasts. Binding of IL‐6 to the IL‐6 receptor of preosteoclasts promotes osteoclast differentiation (Harmer et al., 2019). IL‐6 increases RANKL expression in OBs (Wu et al., 2017). Furthermore, IL‐6/STAT3 signaling affects IL‐1β, prostaglandin E2 (PGE2), and parathyroid hormone related protein (PTHrP), which are related to osteoclast differentiation (Harmer et al., 2019; Hashizume et al., 2008). Treatment with VAE could regulate the IL‐6/STAT3 axis and affect bone metabolism in OVX‐induced OP. However, mechanistic studies including various signaling molecules and transcription factors are necessary.

## 4 CONCLUSION

The results of the present study provide evidence that VAE may be used in the treatment of OA and OP. In the MIA‐induced OA model, VAE treatment effectively protected articular cartilage from degeneration and necrosis, leading to the preservation of chondrocytes and ECM in the cartilage. Protective effects against OA were observed not only in the cartilage but also in the synovium and subchondral bone. In the OVX‐induced OP model, VAE administration improved BMC and BMD. It also recovered trabecular thickness and trabecular bone volume/tissue volume. Taken together, these results demonstrated that VAE treatment improved the clinical symptoms of OA and OP. Thus, VAE might be a potential candidate for OA and OP treatment. However, more studies are needed.

## CONFLICT OF INTEREST

The authors declare no conflicts of interest.

## Supporting information

TableS1
