# Supplementary material for: Beneficial effects of Vigna angularis extract in osteoporosis and osteoarthritis
Source: Food Sci Nutr. 2020 Oct 20;8(12):6550–6. doi: 10.1002/fsn3.1944 (PMC7723184; doi:10.1002/fsn3.1944)
Supplement: Supplementary file 1 — TableS1 [file FSN3-8-6550-s001.docx]

**Supplementary Material**

**Beneficial effects of *Vigna angularis* extract in osteoporosis and osteoarthritis**

**Hyung Jin Lim,^a†^ Sang-Ik Park,^c†^ Seon Gyeong Bak,^ad^ Soyoung Lee,^a^ Young-Bin Baek,^c^ Chang-Min Lee,^c^ Kang Min Lee,^b^ Seung Woong Lee,^a^* Seung-Jae Lee,^a^* and Mun-Chual Rho^a^**

^a^ Immunoregulatory Material Research Center, Korea Research Institute of Bioscience and Biotechnology, Jeongeup-si, 56212, Korea^;^

^b^ Department of Bioactive Material Sciences, Chonbuk National University, Jeonju-si, 54896, Korea

^c^ Laboratory of Veterinary Pathology, College of Veterinary Medicine, Chonnam National University, Gwangju-si, 61186, Korea

^d^ Department of Marine Bio Food Science, Chonnam National University, Korea, Yeosu-si, 59626, Korea

^*^Correspondence and requests for materials should be addressed to S.W.L (email: lswdoc@kribb.re.kr) or S.-J.L (email: seung99@kribb.re.kr)

| **Contents** | | **Pages** |
| --- | --- | --- |
| **Table S1.** | Histopathologic score | S2 |

**Table S1.** Histopathologic score

|  |  | Post OA induction | | | |
| --- | --- | --- | --- | --- | --- |
| Observation | Score | NC | No drug | 50 mg/kg/day | 100 mg/kg/day |
| Chondrocyte loss | + | 0/3 | 0/3 | 1/3 | 2/3 |
|  | ++ | 0/3 | 1/3 | 2/3 | 1/3 |
|  | +++ | 0/3 | 2/3 | 0/3 | 0/3 |
| **Average pathology score** |  | **0** | **2.67** | **1.67** | **1.33** |
| Chondrocyte cloning and hypertrophy | + | 0/3 | 0/3 | 1/3 | 2/3 |
|  | ++ | 0/3 | 0/3 | 1/3 | 1/3 |
|  | +++ | 0/3 | 3/3 | 1/3 | 0/3 |
| **Average pathology score** |  | **0** | **3** | **2** | **1.33** |
| Chondrocyte disorganization | + | 0/3 | 0/3 | 2/3 | 3/3 |
|  | ++ | 0/3 | 2/3 | 1/3 | 0/3 |
|  | +++ | 0/3 | 1/3 | 0/3 | 0/3 |
| **Average pathology score** |  | **0** | **2.33** | **1.33** | **1** |
| Surface irregularity of articular cartilage | + | 0/3 | 1/3 | 2/3 | 3/3 |
|  | ++ | 0/3 | 1/3 | 1/3 | 0/3 |
|  | +++ | 0/3 | 1/3 | 0/3 | 0/3 |
| **Average pathology score** |  | **0** | **2** | **1.33** | **1** |
| Safranin O stain reduction | + | 0/3 | 0/3 | 1/3 | 2/3 |
|  | ++ | 0/3 | 0/3 | 2/3 | 1/3 |
|  | +++ | 0/3 | 3/3 | 0/3 | 0/3 |
| **Average pathology score** |  | **0** | **3** | **1.67** | **1.33** |
| Degeneration/necrosis | + | 0/3 | 0/3 | 2/3 | 2/3 |
|  | ++ | 0/3 | 1/3 | 1/3 | 1/3 |
|  | +++ | 0/3 | 2/3 | 0/3 | 0/3 |
| **Average pathology score** |  | **0** | **2.67** | **1.33** | **1.33** |
| Marginal osteophyte formation | + | 0/3 | 1/3 | 1/3 | 0/3 |
|  | ++ | 0/3 | 1/3 | 0/3 | 0/3 |
|  | +++ | 0/3 | 0/3 | 0/3 | 0/3 |
| **Average pathology score** |  | **0** | **1** | **0.33** | **0** |
| Subchondral changes | + | 0/3 | 0/3 | 1/3 | 2/3 |
|  | ++ | 0/3 | 2/3 | 1/3 | 0/3 |
|  | +++ | 0/3 | 1/3 | 0/3 | 0/3 |
| **Average pathology score** |  | **0** | **2.33** | **1** | **0.67** |
| Fibrillation of cartilage surface | + | 0/3 | 1/3 | 1/3 | 0/3 |
|  | ++ | 0/3 | 1/3 | 0/3 | 0/3 |
|  | +++ | 0/3 | 0/3 | 0/3 | 0/3 |
| **Average pathology score** |  | **0** | **1** | **0.33** | **0** |
| **Total averages pathology score ± S.E.** | | **0** | **2.00 ± 0.078** | **1.10 ± 0.035** | **0.80 ± 0.047** |
